# Tumoral calcinosis complicating CKD-MBD in hemodialysis: a case report

**DOI:** 10.1515/biol-2025-1237

**Published:** 2025-12-30

**Authors:** Chen Chen, Xin Wang, Bi-Hu Gao

**Affiliations:** Department of Nephrology, The Zhongshan Hospital Affiliated to Dalian University, Dalian, 116001, Liaoning, China

**Keywords:** chronic kidney disease-mineral and bone disorder, ectopic calcification, hemodialysis, tumoral calcinosis

## Abstract

Chronic kidney disease–mineral and bone disorder (CKD-MBD) is a common complication in patients with chronic kidney failure and is frequently associated with ectopic calcification. One of its less frequent manifestations, tumoral calcinosis (TC), involves periarticular soft tissue deposition of calcium-phosphate complexes and may develop in patients undergoing maintenance dialysis for end-stage renal disease. TC contributes to significant morbidity, including pain and functional impairment, and may be associated with increased mortality. This report describes a patient receiving maintenance hemodialysis who developed calcified periarticular masses in the left hip and right shoulder. The diagnosis of secondary TC related to CKD-MBD was established based on laboratory parameters, radiographic imaging, and histopathological evaluation. Management included surgical excision of the lesions, dietary phosphate restriction, administration of non-calcium-based phosphate binders, use of low-calcium dialysate, and initiation of high-flux hemodialysis. These therapeutic measures were associated with gradual reduction in lesion size and symptomatic improvement.

## 1 Introduction

Chronic kidney disease–mineral and bone disorder (CKD-MBD) is a frequent complication of chronic renal failure (CRF), particularly among patients receiving maintenance hemodialysis (MHD). One of its clinical manifestations is ectopic calcification, which may present in various forms, including vascular calcification, cutaneous deposits, and periarticular soft tissue involvement. Although relatively uncommon, periarticular soft tissue calcification has been documented in patients with end-stage renal disease (ESRD) undergoing dialysis and is referred to in the literature as tumoral calcinosis (TC) [1].

TC is characterized by the deposition of calcium (Ca) salts in soft tissues adjacent to large joints. While its exact pathogenesis remains unclear, several contributing mechanisms have been proposed, including: (1) abnormalities in serum calcium, phosphate, parathyroid hormone, vitamin D, and lipid metabolism [2]; (2) reactive calcification of periarticular collagen fibers in response to chronic local stimulation; (3) genetic predisposition, such as autosomal recessive inheritance associated with fibroblast growth factor 23 deficiency, resulting in familial hyperphosphatemic tumoral calcinosis (HFTC); and (4) localized mechanical factors, such as repeated microtrauma leading to impaired tissue perfusion [3].

Severe ectopic calcification can significantly impair a patient’s quality of life and, in advanced cases, may lead to life-threatening complications. This report describes the clinical presentation, diagnosis, and management of a patient on MHD who developed TC, followed by a review of relevant literature from both Chinese and international sources.

## 2 Clinical data

A 54-year-old woman was admitted in November 2023 with an eight-year history of abnormal renal function and a recent onset of right shoulder joint pain of three days’ duration. In 2015, during a routine physical examination at Wafangdian Central Hospital, her serum creatinine level was 110 μmol/L. Urinalysis revealed proteinuria (3+) and occult hematuria (2+). No formal medical treatment was initiated; instead, the patient self-administered traditional Chinese herbal remedies, though the specific regimen was not documented. Serial laboratory testing demonstrated progressive worsening of renal function. One month later, she developed recurrent nausea, vomiting, and anorexia, with serum creatinine reaching approximately 700 μmol/L. A diagnosis of stage 5 chronic kidney disease (CKD) was made. A right forearm arteriovenous fistula was created at a local hospital, and maintenance hemodialysis (three sessions per week) was initiated following fistula maturation and continued thereafter.

In 2017, the patient developed a mass over the left hip with mild tenderness and limited range of motion, without erythema or swelling. No medical intervention was undertaken at that time. By 2019, mass lesions were present in both the left hip and right shoulder. Laboratory evaluation revealed serum calcium 2.63 mmol/L, phosphorus (P) 2.15 mmol/L, and intact parathyroid hormone (iPTH) 1,282 pg/mL. A diagnosis of secondary hyperparathyroidism was established. The patient underwent two parathyroidectomies in April and July 2020. Postoperatively, the patient did not follow dietary phosphate restrictions but continued regular use of calcitriol and Caltrate D. Serum calcium, phosphorus, and iPTH levels were monitored every three months, with dosage adjustments made accordingly. Calcium acetate was taken intermittently.

**Figure 1:**
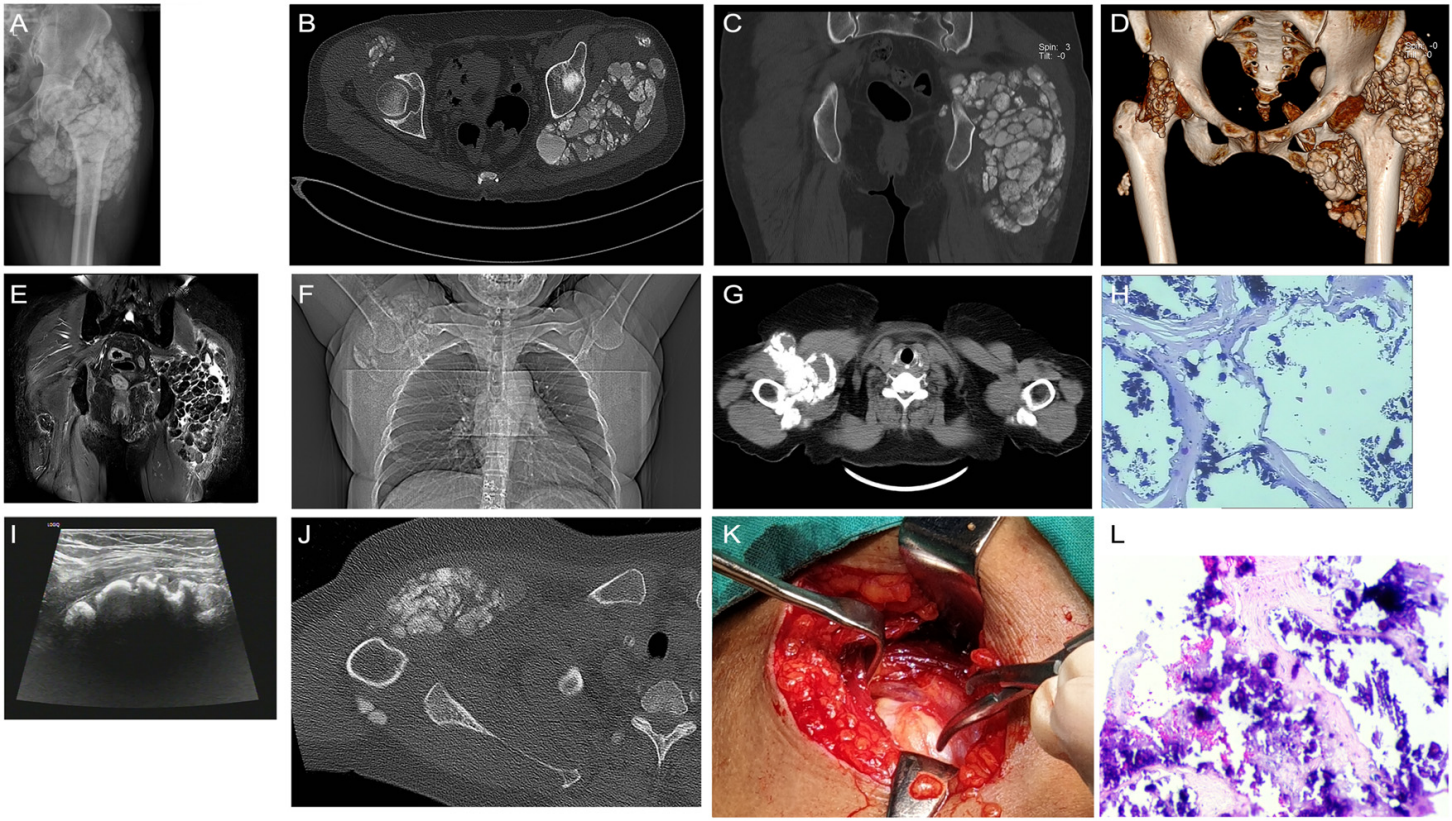
Multisite periarticular soft tissue calcification in a patient receiving hemodialysis for CKD–MBD. A: Anteroposterior radiograph of the left hip showing periarticular soft tissue calcification. B–D: Non-contrast CT with three-dimensional reconstruction demonstrating multiple calcified foci in the periarticular soft tissues of the left hip. E: Pelvic MRI showing abnormal signal intensities consistent with soft tissue calcification and a cystic lesion within the left gluteus medius. F–G: Chest CT images showing multiple calcified soft tissue nodules in the right axillary region adjacent to the shoulder joint. H: Intraoperative histopathology of the excised left gluteal mass showing metastatic calcification (hematoxylin and eosin staining, × 10 magnification). I: Color Doppler ultrasound revealing multiple calcified foci in the subcutaneous tissue of the right anterior axilla. J: Non-contrast CT of the right shoulder demonstrating soft tissue calcifications surrounding the joint and anterior to the proximal humerus. K: Intraoperative appearance of the excised right shoulder mass, with visible white, sand-like material. L: Histopathological section of the right shoulder mass showing calcification within fibrous connective tissue; no multinucleated giant cells, chronic inflammatory infiltrates, or necrosis were observed (hematoxylin and eosin staining, × 10 magnification).

In August 2021, the patient reported recurrent generalized bone pain and restricted mobility of the left hip. Anteroposterior and lateral radiographs of the left hip revealed periarticular lesions (Figure 1A). A non-contrast computed tomography (CT) scan with three-dimensional reconstruction revealed high-density foci within the periarticular soft tissues, consistent with soft tissue calcification secondary to renal osteodystrophy (Figure 1B–D). Pelvic magnetic resonance imaging (MRI) showed abnormal signal intensities in the periarticular soft tissues and musculature of both hips, with a cystic lesion identified within the left gluteus medius (Figure 1E). Chest CT revealed multiple calcified soft tissue foci in the right axillary region (Figures 1F–G).

In July 2022, the patient underwent surgical excision of a large mass in the left gluteal region. Intraoperative histopathological evaluation confirmed metastatic calcification in the left hip area (Figure 1H). Three days prior to the most recent hospital admission, she developed acute right shoulder pain accompanied by reduced joint mobility, localized tenderness, and a palpable mass. There were no associated symptoms such as fever, chills, dizziness, headache, chest discomfort, abdominal pain, or abdominal distension. The patient reported preserved appetite and sleep, anuric status, and normal bowel movements. She was admitted for further diagnostic evaluation and management.

The patient had a seven-year history of hypertension, with a documented maximum blood pressure of 180/100 mmHg. Blood pressure control was suboptimal due to irregular adherence to antihypertensive therapy. There was no history of diabetes mellitus, coronary artery disease, gout, bone fractures, or traumatic injury.

On physical examination, vital signs were as follows: temperature 36.8 °C, pulse 86 beats per minute, respiratory rate 18 breaths per minute, and blood pressure 142/95 mmHg. The patient appeared chronically ill, with moderate nutritional status and clinical signs of anemia. No skin ulcerations or gangrenous changes were observed. Cardiac, pulmonary, and abdominal examinations were unremarkable. There was no percussion tenderness over the renal angles, costovertebral angle tenderness, spinal deformity, or vertebral tenderness. A firm, tender mass approximately 10 × 10 cm in size was palpable in the periarticular region of the right shoulder. The overlying skin was intact, without erythema or warmth. Shoulder joint mobility was restricted.

### 2.1 Auxiliary examinations on admission

Complete blood count: White blood cell count (WBC): 7.0 × 10^9^/L; neutrophils (*N*): 70.6 %; hemoglobin (Hb): 112 g/L.

Biochemistry: Serum creatinine: 797.7 μmol/L; serum albumin: 44.9 g/L.

Electrolytes: Serum calcium (Ca): 2.40 mmol/L (corrected Ca: 2.30 mmol/L); phosphorous: 1.63 mmol/L; calcium–phosphate product: 46.49 mg/dl.

Other markers: Alkaline phosphatase: 48 IU/L; iPTH: 1.85 pg/mL.

Imaging studies: Ultrasound of the right anterior axillary region revealed multiple calcified foci within the subcutaneous soft tissues (Figure 1I). Non-contrast CT of the right scapular region demonstrated multiple calcified foci within the soft tissues surrounding the right shoulder and anterior to the proximal right humerus. These findings, when interpreted in conjunction with the patient’s clinical history and biochemical abnormalities, were consistent with disordered mineral and bone metabolism. Clinical correlation and follow-up imaging were recommended (Figure 1J).

### 2.2 Final clinical diagnosis

– CKD stage 5 due to chronic glomerulonephritis
– ESRD requiring maintenance hemodialysis
– Anemia secondary to CKD
– Hyperphosphatemia
– Secondary hyperparathyroidism
– CKD-MBD
– Tumoral calcinosis
– Grade 3 hypertension

Following admission, the patient continued MHD three times per week using a dialysate containing 1.25 mmol/L of calcium. Additional medical management included antihypertensive therapy, correction of anemia, and phosphate-lowering treatment. In November 2023, surgical excision of the periarticular mass in the right shoulder was performed. Intraoperative histopathological examination revealed fibrous connective tissue with extensive calcification (Figure 1K–L).

Postoperative recommendations included dietary restriction of high-phosphorus foods, optimization of dialysis adequacy, and regular administration of non-calcium-based phosphate binders. Gradual resolution of bone pain was observed during the follow-up period (Table 1).

**Table 1:** Clinical course of the patient.

Time points	Brief description of the condition
2015–08	The blood creatinine level was found to be 110 umol/L, with urine protein at 3+ and urine occult blood at 2+. The patient was taking traditional Chinese medicine on their own (the specific treatment duration is unknown). One month later, the blood creatinine level was approximately 700 umol/L. A right forearm arteriovenous fistula formation surgery was performed, and regular hemodialysis treatment was carried out three times a week that year.
2017–05	A mass was found in the left hip joint, accompanied by mild tenderness upon pressure, poor mobility, no redness or swelling, and no special treatment was given.
2019–06	There were masses formed in the left hip joint and the right shoulder joint. The blood calcium level was 2.63 mmol/L, the blood phosphorus level was 2.15 mmol/L, and the iPTH level was 1,282 pg/ml.
2020-04/07	Had two “parathyroidectomy” procedures. Postoperative diet was not controlled. Continued to take calcitriol and calcium carbonate D. Regularly monitored Ca, P and iPTH every 3 months, and adjusted the drug dosage according to the test results. Occasionally took calcium acetate.
2021–08	The patient experienced another episode of generalized bone pain, accompanied by limited movement of the left hip joint. Complete left hip joint anteroposterior and lateral radiographs, dual hip joint CT plain scan + three-dimensional reconstruction, pelvic soft tissue MRI, and chest CT examinations were performed.
2022–07	The patient underwent removal of a large tumor in the left buttock.
2023–11	Pain in the right shoulder joint, accompanied by limited movement, and a palpable mass could be felt. There was also a tender sensation in the local area. Laboratory tests: WBC 7.0 × 109/L, *N* 70.6 %, Hb 112 g/L; serum Cr 797.7 mol/L; serum albumin 44.9 g/L; electrolytes: Ca 2.40 mmol/L (corrected Ca 2.30 mmol/L), P 1.63 mmol/L, Ca × P = 46.49 mg/dl. Serum alkaline phosphatase 48 IU/L; iPTH 1.85 pg/ml. Comprehensive examination of superficial soft tissue masses by ultrasound and CT plain scan of the right scapula was conducted. Three times of hemodialysis were performed every week. In November 2023, “removal of the tumor around the right shoulder joint” was performed.

**Informed consent:** Informed consent has been obtained from all individuals included in this study.

**Ethical approval:** The research related to human use has been complied with all the relevant national regulations, institutional policies and in accordance with the tenets of the Helsinki Declaration, and has been approved by the Ethics Committee of the Zhongshan Hospital Affiliated to Dalian University (No.BLBD 2025–006).

## 3 Discussion

Ectopic calcification is a relatively common complication in patients with CRF and CKD-MBD [4]. Common sites of calcification include vascular walls, cardiac valves, and subcutaneous tissues, which may contribute to arteriosclerosis, cardiac dysfunction, and skin necrosis. These complications collectively impair quality of life and increase mortality risk [2]. In contrast, TC is a less frequent manifestation in patients receiving MHD, with a reported incidence ranging from 0.5 % to 3 % [5].

Diagnosis of TC relies on clinical history and characteristic imaging features. Radiographic and cross-sectional imaging typically reveal irregular, lobulated, or mass-like aggregations of calcified nodules of varying sizes, commonly located near extensor surfaces of large joints. In advanced stages, a “streaming” or sedimentation appearance may be seen, while adjacent bones and joints are usually spared. Histopathological analysis typically demonstrates amorphous calcium salt deposition within fibrotic stroma, accompanied by multinucleated giant cells and chronic inflammatory cell infiltration [5]. TC most frequently affects the shoulders, hips, and elbows, but may also involve the fingers and toes, leading to joint swelling, pain, and restricted mobility [6], 7]. In the present case, the patient developed multifocal periarticular calcification with symptoms affecting the shoulders, hips, and axillary regions. Despite surgical resection, recurrence of symptoms was observed.

Management of TC in dialysis patients includes both conservative and surgical approaches. Conservative treatment typically consists of optimizing dialysis adequacy, restricting dietary calcium and phosphorus intake, and administering non-calcium-based phosphate binders to control hyperphosphatemia. Active vitamin D analogues may be used to suppress parathyroid hormone (PTH) levels, while low-calcium dialysate helps mitigate calcium overload. In cases of persistent hyperparathyroidism with hypercalcemia, calcium-sensing receptor agonists such as cinacalcet may be employed. Surgical parathyroidectomy is considered when conservative measures are ineffective. Several studies have demonstrated a significant reduction in ectopic calcification following parathyroidectomy [1].

Additional pharmacologic agents include sodium thiosulfate, which has antioxidant properties and may inhibit ectopic calcification while promoting dissolution of existing deposits [8]. Bisphosphonates, as pyrophosphate analogues, inhibit osteoclastic activity and may be appropriate in early CKD-related bone disease when estimated glomerular filtration rate exceeds 35 mL/min/1.73 m^2^. However, data supporting their use in advanced CKD remain limited, primarily due to concerns regarding nephrotoxicity [9]. Vitamin K also plays a role in inhibiting vascular calcification through its function as a cofactor in the carboxylation of matrix Gla protein, a key regulator of vascular and bone mineralization [10].

In the present case, several factors likely contributed to the development of TC. Persistent hyperphosphatemia was attributed to inadequate dietary phosphate restriction and inconsistent use of phosphate binders, leading to secondary hyperparathyroidism, increased bone resorption, and elevation of the calcium–phosphorus product. Despite thrice-weekly hemodialysis, the patient’s Kt/V (dialyzer clearance of urea×dialysis time÷volume of distribution of urea) values were frequently below 1.2, indicating suboptimal dialysis adequacy. In addition, prolonged administration of active vitamin D resulted in sustained PTH suppression, increasing the risk of hypercalcemia and facilitating ectopic calcification [11].

Ultrasonographic findings of parathyroid hyperplasia, in conjunction with soft tissue calcification around the right shoulder, supported the decision to proceed with surgical excision. Concurrent conservative measures included dietary phosphate restriction, administration of non-calcium-based phosphate binders, use of low-calcium dialysate, and initiation of high-flux hemodialysis. This combined approach was associated with gradual regression of the calcified masses and improvement in clinical symptoms, without the need to increase dialysis frequency.

Zhong et al. [12] reported a similar case of CKD complicated by TC, in which the patient was diagnosed with tertiary hyperparathyroidism following multidisciplinary evaluation. They concluded that surgical excision alone was prone to recurrence and emphasized parathyroidectomy as a primary treatment. The recurrence pattern observed in our case was consistent with this finding. However, the addition of comprehensive conservative therapy in our patient postoperatively contributed to a favorable outcome, despite the severity of calcification.

Hu et al. [13] described a patient with ESRD and multiple sites of ectopic calcification who showed marked improvement following intensified automated peritoneal dialysis combined with icodextrin, cinacalcet, phosphate binders, calcitriol, and nutritional support. Similarly, Berrio et al. [14] highlighted the importance of a comprehensive therapeutic strategy for managing mineral and bone disorder in patients undergoing dialysis to prevent progression of ectopic calcification.

Although the multimodal treatment approach in this case yielded favorable clinical outcomes, further investigation involving larger patient cohorts is necessary to evaluate its generalizability and efficacy in broader clinical practice.

## References

[1] Sunder S, Verma H, Venkataramanan K (2013). Cervical tumoral calcinosis with secondary hyperparathyroidism in a chronic hemodialysis patient. Hemodial Int.

[2] Düsing P, Zietzer A, Goody PR, Hosen MR, Kurts C, Nickenig G (2021). Vascular pathologies in chronic kidney disease: pathophysiological mechanisms and novel therapeutic approaches. J Mol Med Berl.

[3] Bartolomeo K, Tan XY, Fatica R (2022). Extraosseous calcification in kidney disease. Cleve Clin J Med.

[4] Xu SS, Hao LH, Guan YM (2024). Reversal of complete atrioventricular block in dialysis patients following parathyroidectomy: a case report. World J Clin Cases.

[5] Olejarz M, Szczepanek-Parulska E, Antosik P, Ostałowska-Klockiewicz A, Ruchała M (2022). Tumoral calcinosis in the periarticular soft tissue of the left shoulder joint secondary to end-stage renal disease. Pol Arch Intern Med.

[6] Yano H, Kinjo M (2021). Tumoral calcinosis. Cleve Clin J Med.

[7] He L, Li M, Lin C, Yan K, Yang C, Tang J (2023). Multiple uremic tumoral calcinosis in periarticular soft tissues with chronic renal failure: a case report. Front Endocrinol.

[8] Gauffenic A, Ratsimbazafy V, Ostertag A, Linglart A, Bourrat E, Leroy C (2023). Effectiveness of topical sodium thiosulfate for ectopic calcifications and ossifications. Results of the CATSS-O study. Semin Arthritis Rheum.

[9] Hildebrand S, Cunningham J (2021). Is there a role for bisphosphonates in vascular calcification in chronic kidney disease?. Bone.

[10] Fusaro M, Gallieni M, Porta C, Nickolas TL, Khairallah P (2020). Vitamin K effects in human health: new insights beyond bone and cardiovascular health. J Nephrol.

[11] Patil C, Lavanya P, Mangalagiri N, Bommanagari D, Gupta P (2025). Pulmonary metastatic calcifications secondary to chronic renal failure. Cureus.

[12] Zhong M, Yan SJ, Huang LN (2024). Tumoral calcinosis in a patient with chronic kidney disease: a case report. Asian J Surg.

[13] Hu S, Zhang Z, Sun D, Chen Y, Wang N, Xu T (2025). Multiple ectopic calcifications in end-stage renal disease: role of inflammation and partial reversibility with intensified peritoneal dialysis-a case report. BMC Nephrol.

[14] Moral Berrio E, Cox Conforme RA, Elías R, De La Flor JC, Rodríguez Tudero C, Sánchez de la Nieta-García MD (2025). An unusual case of uremic tumoral calcinosis with atypical manifestation in a patient on peritoneal dialysis: case report and review of the literature. Med Sci.

